# A step-by-step approach to improve data quality when using commercial business lists to characterize retail food environments

**DOI:** 10.1186/s13104-016-2355-1

**Published:** 2017-01-07

**Authors:** Kelly K. Jones, Shannon N. Zenk, Elizabeth Tarlov, Lisa M. Powell, Stephen A. Matthews, Irina Horoi

**Affiliations:** 1 Department of Health Systems Science, College of Nursing, University of Illinois at Chicago, 845 S. Damen Ave, Chicago, IL 60612 USA; 2 Center of Innovation for Complex Chronic Healthcare, Edward Hines, Jr. VA Hospital, Hines, IL 60141 USA; 3 Health Policy and Administration Division, School of Public Health, University of Illinois at Chicago, 1603 W. Taylor St, Chicago, IL 60612 USA; 4 Department of Sociology and Criminology, The Pennsylvania State University, 206 Oswald Tower, University Park, PA 16802 USA; 5 Department of Anthropology, The Pennsylvania State University, 410 Carpenter Building, University Park, PA 16802 USA; 6 Department of Economics, University of Illinois at Chicago, 601 S. Morgan St, Chicago, IL 60607 USA

**Keywords:** Business lists, InfoUSA, Dun and Bradstreet, Neighborhood food environment

## Abstract

**Background:**

Food environment characterization in health studies often requires data on the location of food stores and restaurants. While commercial business lists are commonly used as data sources for such studies, current literature provides little guidance on how to use validation study results to make decisions on which commercial business list to use and how to maximize the accuracy of those lists. Using data from a retrospective cohort study [Weight And Veterans’ Environments Study (WAVES)], we (a) explain how validity and bias information from existing validation studies (count accuracy, classification accuracy, locational accuracy, as well as potential bias by neighborhood racial/ethnic composition, economic characteristics, and urbanicity) were used to determine which commercial business listing to purchase for retail food outlet data and (b) describe the methods used to maximize the quality of the data and results of this approach.

**Methods:**

We developed data improvement methods based on existing validation studies. These methods included purchasing records from commercial business lists (InfoUSA and Dun and Bradstreet) based on store/restaurant names as well as standard industrial classification (SIC) codes, reclassifying records by store type, improving geographic accuracy of records, and deduplicating records. We examined the impact of these procedures on food outlet counts in US census tracts.

**Results:**

After cleaning and deduplicating, our strategy resulted in a 17.5% reduction in the count of food stores that were valid from those purchased from InfoUSA and 5.6% reduction in valid counts of restaurants purchased from Dun and Bradstreet. Locational accuracy was improved for 7.5% of records by applying street addresses of subsequent years to records with post-office (PO) box addresses. In total, up to 83% of US census tracts annually experienced a change (either positive or negative) in the count of retail food outlets between the initial purchase and the final dataset.

**Discussion:**

Our study provides a step-by-step approach to purchase and process business list data obtained from commercial vendors. The approach can be followed by studies of any size, including those with datasets too large to process each record by hand and will promote consistency in characterization of the retail food environment across studies.

**Electronic supplementary material:**

The online version of this article (doi:10.1186/s13104-016-2355-1) contains supplementary material, which is available to authorized users.

## Background

Identifying contributions of the neighborhood food environment to diet and related health outcomes is of considerable interest (e.g., [1–4]). Information on the type and location of retail food outlets is central to this research [5]. A variety of primary and secondary data sources, including in-person audits, government sources, phone books/yellow pages, and commercial business lists [6], have been used to identify local businesses and classify them by type. Each source has strengths and limitations related to ease of acquisition, cost, geographic coverage, and validity [6–11]. Determination of the optimal source requires weighing these factors in the context of the underlying research question.

Primary data collection in the form of in-person audits are widely considered to be the gold standard but the costs of in-person data collection can be very high [6, 10] and thus the geographic area it is possible to cover with this approach is small without substantial financial investment. Additionally, in-person audit data cannot be collected retrospectively. Administrative records generated by the taxing (e.g., alcohol and food), licensing (e.g., restaurant health inspections), and programmatic activities (e.g., databases of Supplemental Nutrition Assistance Program authorized retailers) of local and state government agencies are also sources for secondary retail food environment data [6, 11]. However, the information, collected to fulfill administrative or regulatory requirements, may not match the research need in terms of specificity (e.g., differentiating between types of establishments) and other properties. Further, combining data from multiple sources or across administrative areas can be complicated by differences in laws, regulations and ordinances (both across time and administrative area) resulting in dissimilarities in the specific information collected as well as differences in database design. Freedom of information requests can make it possible to obtain governmental data, but the complexity grows as the number of covered administrative areas requiring requests increases [12, 13]. Furthermore, because there are so many unique sources for government data, validation studies have shown results ranging from fair to almost perfect [6]. Another secondary source for retail food outlet data is phone books/yellow pages [6, 11]. Compiling and entering data from phone books/yellow pages across multiple administrative areas can be costly and challenging depending on the study scope, and validation studies have shown wide variability in data quality, with Fleischhacker et al. [6] reporting fair to almost perfect validity.

Commercial business lists are compilations of information about businesses collected and maintained for marketing purposes. While purchasing commercial business lists can require a significant financial investment and the researcher lacks control over data collection processes and quality, they reduce many of the difficulties associated with other options [6, 14–18]. For example, they are available for historic time periods and across administrative areas, classify retail food outlets according to common classification systems (i.e., Standard Industrial Classification (SIC) or North American Industry Classification System), have uniform rules for data collection that do not vary across administrative areas, and are provided in a pre-established and organized database format with available metadata and documentation. Because of these advantages, commercial business lists are often the data source of choice for retail food environment studies that are retrospective, cover a large geographic footprint, or examine a broad set of store types [19]. Additionally, validation studies using in-person audits as the gold standard show criterion-related validity for commercial business lists that is as good as or better than government sources and phone books/yellow pages, with reported validation statistics of moderate to almost perfect [6].

Two of the most widely used commercial business lists for food-related outlets in health research are InfoUSA (or ReferenceUSA, both divisions of InfoGroup, Inc.) and Dun and Bradstreet [17, 19]. Both companies provide detailed information about individual businesses, including business type, size, and location, and the ability to track businesses through time. However, neither company provides a completely accurate census of businesses [6]. For example, in lists from either company, some stores that actually exist are missing while others that have closed are listed. Therefore, when choosing to use commercial business lists, decisions about purchasing and processing must be made.

Liese et al. [15] recommend purchasing business list data from multiple companies and combining for the most complete and accurate representation of the retail food environment, and at least one prior study reported on a strategy for merging two purchased lists [20]. Combining lists, however, may be infeasible due to the study scope (e.g., multiple years of data, large geographic footprint) and limited resources (e.g., financial resources to purchase multiple lists, personnel resources to clean, merge, and deduplicate multiple lists). When only one commercial business list can be purchased, a variety of different aspects of validity and bias should be considered and interpreted in the context of the study goals, including validity related to classification of outlet type, location, and systematic bias. Fortunately, a number of business list data validation studies have been conducted. However, little guidance is available on how best to use results from those studies to inform decisions about data selection and optimize data quality.

To address this gap, we developed a step-by-step approach to improve data quality when using commercial business list data to characterize the retail food environment. In this paper, we illustrate this approach, which involved two major phases, using the Weight and Veterans’ Environment Study (WAVES), a nationwide, longitudinal study of neighborhood environments and body weight status. First we discuss the use of results from previously-conducted validation studies to select between commercial business list sources (InfoUSA and Dun and Bradstreet). Second, we describe strategies to maximize the quality of the purchased data. Finally, we provide results of the data maximization strategies. We begin with an overview of WAVES.

### Overview of WAVES

WAVES is a retrospective cohort study of diet- and physical activity-related attributes of adults’ residential environments and their longitudinal relationships with body weight, metabolic risk (e.g., blood pressure, serum lipids, serum glucose), and engagement in and outcomes of a nationwide weight management program. WAVES links information on the retail food environment through veterans’ residential addresses to individual health information for each year 2009 through 2015, including spatial accessibility of several types of food stores and restaurants. The study focuses on 3.2 million US military veterans receiving Department of Veterans Affairs (VA) health care, including those enrolled in the VA’s nationwide weight management program, MOVE! [21]. The overarching hypothesis of WAVES is that environments with more facilitating attributes help people maintain a healthier body weight and metabolic risk status and achieve better weight management program outcomes.

This study presented several challenges in characterizing the retail food environment, not uncommon in this area of research. We required both contemporary and historical data covering the entire continental United States for multiple years for a wide variety of retail food outlet types, including supermarkets, grocery stores, convenience stores, pharmacies, liquor stores, general merchandise stores, and limited service restaurants. For these reasons, commercial business list data was deemed the best option. Because the resources that would be required to purchase and then combine (and deduplicate) data from more than one company were not available, we reviewed several validation studies to guide our decision about which company’s data to purchase.

## Methods

### Comparison of previous validation studies

When determining which commercial business list (InfoUSA or Dun and Bradstreet) to purchase for each retail food outlet type, we were concerned about three types of validity including count, classification, and locational, as well as bias by neighborhood characteristics. (see Table 1 for definitions of validity terms). Thus, we reviewed validation studies that (1) included both InfoUSA (as either InfoUSA or ReferenceUSA) and Dun and Bradstreet data; (2) used in-person audits as the gold standard comparison; (3) were conducted in the United States; and (4) calculated validity statistics based on the exact location of each establishment, rather than presence within an administrative area. Five validation studies and one systematic review were identified [6, 8, 15–17, 22, 23]. Below we summarize and evaluate the findings of these studies in regard to each data source’s count, classification, and locational accuracy. Within each of these categories we considered bias by neighborhood racial/ethnic composition, economic characteristics, and urbanicity.

**Table 1:** Definitions of validity terms

Term	Definition
Count accuracy	Number of outlets is neither an under- nor over-count
Classification accuracy	Business type is correctly identified
Locational accuracy	Geographic coordinates are accurate within an acceptable level of precision
True positive (TP)	Outlet present in business list and observed on the ground
False positive (FP)	Outlet present in business list and not observed on the ground
True negative (TN)	Outlet not present in business list and not observed on the ground
False negative (FN)	Outlet not present in business list and observed on the ground
Sensitivity	Proportion of observed outlets that are included in the business list: (TP)/(TP + FN)
Positive predictive value (PPV)	Likelihood that an outlet present in the business list is observed: (TP)/(TP + FP)
Concordance	Proportion of outlets both present in the business list and observed out of all outlets either in the business list or observed: (TP)/(TP + FP + FN)

#### Count accuracy

The first key factor we considered was the count accuracy of each business list, meaning that we sought data with optimal sensitivity and positive predictive value (PPV) for food stores and restaurants. High sensitivity results indicate that there is not excessive undercount in the data source, and high PPV results indicates that there is not excessive overcount. In side-by-side comparisons between InfoUSA and Dun and Bradstreet, two of three studies found InfoUSA had better overall sensitivity for food stores and three of three concluded InfoUSA had better overall sensitivity for restaurants [8, 15, 17, 22]. One of three studies found overall PPV was better for InfoUSA for food stores, and all three studies showed InfoUSA had better PPV than Dun and Bradstreet for restaurants [8, 15, 17, 22] (Table 2). Four studies examined bias in count accuracy for some food store types and restaurants, and two found differences by racial/ethnic composition [16, 17], two by economic characteristics [16, 17], and two by urbanicity [15, 17] without clear patterns suggesting more bias for one data source than another (Table 3).

**Table 2:** Identification of data source (InfoUSA or Dun and Bradstreet) with better count accuracy statistics for food stores and restaurants

**Study**	All outlets		All food stores		All restaurants	
	Sensitivity	PPV	Sensitivity	PPV	Sensitivity	PPV
D’Angelo [22] estimate (SE)	N/A	N/A	*InfoUSA: 0.84 (0.01)*	InfoUSA: 0.87 (0.01)	N/A	N/A
			Dun and Bradstreet: 0.64 (0.02)	Dun and Bradstreet: 0.91 (0.01)		
Fleischhacker [8] estimate [CI]	*InfoUSA: 0.89 [0.86, 0.92]*	*InfoUSA: 0.67 [0.63, 0.70]*	N/A	N/A	*InfoUSA: 0.91 [0.88, 0.94]*	*InfoUSA: 0.71 [0.66, 0.75]*
	Dun and Bradstreet: 0.41 [0.37, 0.45]	Dun and Bradstreet: 0.31 [0.28, 0.34]			Dun and Bradstreet: 0.38 [0.32, 0.44]	Dun and Bradstreet: 0.29 [0.25, 0.34]
Liese [15] estimate [CI]	*InfoUSA: 0.65 [0.63, 0.67]*	*InfoUSA: 0.86 [0.85, 0.88]*	InfoUSA: 0.61 [0.58, 0.64]	InfoUSA: 0.82 [0.79, 0.85]	*InfoUSA: 0.67 [0.65, 0.70]*	*InfoUSA: 0.90 [0.88, 0.92]*
	Dun and Bradstreet: 0.55 [0.53, 0.57]	Dun and Bradstreet: 0.78 [0.76, 0.80]	Dun and Bradstreet: 0.63 [0.60, 0.66]	Dun and Bradstreet: 0.76 [0.73, 0.79]	Dun and Bradstreet: 0.50 [0.47, 0.53]	Dun and Bradstreet: 0.79 [0.77, 0.82]
Powell [17] estimate (SE)	N/A	N/A	*InfoUSA: 0.64 (0.02)*	*InfoUSA: 0.61 (0.02)*	*InfoUSA: 0.65 (0.01)*	*InfoUSA: 0.79 (0.01)*
			Dun and Bradstreet: 0.52 (0.02)	Dun and Bradstreet: 0.45 (0.02)	Dun and Bradstreet: 0.55 (0.01)	Dun and Bradstreet: 0.66 (0.01)

Italic indicates statistically higher validity statistic as compared to the other data source

Standard errors and confidence intervals reported as originally reported in the cited papers

**Table 3:** Bias findings from validation studies in InfoUSA and Dun and Bradstreet business lists

Study	Racial/ethnic composition		Economic characteristics		Urbanicity	
	InfoUSA	Dun and Bradstreet	InfoUSA	Dun and Bradstreet	InfoUSA	Dun and Bradstreet
Count accuracy						
Fleischhacker [8]	N/A	N/A	N/A	N/A	No differences found	No differences found
Liese [15]	N/A	N/A	N/A	N/A	Urban areas had highest accuracy of stores. Rural areas had lowest accuracy of stores. Suburban areas had the lowest accuracy of restaurants	Urban areas had highest accuracy for stores and restaurants. Rural areas had lowest accuracy for stores and restaurants
Liese [16]	Majority white neighborhoods had lowest accuracy	No differences found	High income and non-poor neighborhoods had lowest accuracy	No differences found	N/A	N/A
Powell [17]	Majority black neighborhoods had lowest accuracy for food stores and restaurants. Majority non-Hispanic neighborhoods has lower accuracy for food stores	Majority black neighborhoods had lowest accuracy for restaurants and no difference for food stores	No differences found	High income areas had lowest accuracy for food stores and no differences for restaurants	Urban areas had highest accuracy of stores and restaurants. Rural areas had lowest accuracy of stores and restaurants	Urban areas had highest accuracy of stores and restaurants. Rural areas had lowest accuracy of stores and restaurants
Classification accuracy						
Han [23]	Majority non-Hispanic and majority black neighborhoods had lowest classification accuracy	Majority non-Hispanic and majority black neighborhoods had lowest classification accuracy	No differences found	No differences found	N/A	N/A
Locational accuracy						
Liese [15]	N/A	N/A	N/A	N/A	Urban areas were located with the least distance between observed and listed location. Records in suburban areas were most likely to be allocated to the correct census tract	Urban areas were located with the least distance between observed and listed location. Records in suburban areas were most likely to be allocated to the correct census tract

#### Classification accuracy

The second key factor we examined was each source’s accuracy in classifying outlets into store or restaurant types. Both companies provide SIC codes which can be used to classify individual outlets into business type. Three validation studies examined classification accuracy in InfoUSA and Dun and Bradstreet, with mixed results [16, 17, 23]. Liese et al. [17] and Powell et al. [17] both showed that conditioning validity assessment on store or restaurant classification match reduced both sensitivity and PPV. When accounting for classification error, some differences were seen between the datasets in sensitivity or PPV for specific store and restaurant types, with InfoUSA generally outperforming Dun and Bradstreet (Table 4). A notable exception is limited-service restaurants, where both Liese et al. [16] and Powell et al. [17] reported better sensitivity in Dun and Bradstreet. Han et al. [23] found InfoUSA had worse concordance than Dun and Bradstreet for supermarket and grocery store classification, but better concordance for convenience store classification. Powell et al. [17] found InfoUSA had better concordance for both supermarket and grocery stores and convenience stores, but worse concordance for limited service restaurants (Table 4). One study investigated bias in classification accuracy for food stores in neighborhood racial/ethnic composition and economic characteristics and found worse classification accuracy in non-Hispanic and black neighborhoods in both InfoUSA and Dun and Bradstreet [23] (Table 3).

**Table 4:** Identification of business list with better classification accuracy for selected food stores and restaurants

Study	Supermarkets and grocery stores			Convenience stores			Limited service restaurants		
	Sensitivity	PPV	Concordance	Sensitivity	PPV	Concordance	Sensitivity	PPV	Concordance
Han [23] estimate	N/A	N/A	InfoUSA: 69–81%	N/A	N/A	*InfoUSA: 49%*	N/A	N/A	N/A
	N/A	N/A	*Dun and Bradstreet: 75–91%*	N/A	N/A	Dun and Bradstreet: 24%	N/A	N/A	N/A
Liese [16] estimate [CI]	InfoUSA: 0.61 [0.54, 0.69]	*InfoUSA: 0.57 [0.50, 0.65]*	N/A	*InfoUSA: 0.63 [0.59, 0.68]*	*InfoUSA: 0.79 [0.74, 0.83]*	N/A	InfoUSA: 0.08 [0.06, 0.10]	InfoUSA: 0.81 [0.71, 0.91]	N/A
	Dun and Bradstreet: 0.58 [0.51, 0.66]	Dun and Bradstreet: 0.39 [0.33, 0.46]	N/A	Dun and Bradstreet: 0.40 [0.36, 0.45]	Dun and Bradstreet: 0.63 [0.58, 0.69]	N/A	*Dun and Bradstreet: 0.41 [0.38, 0.45]*	Dun and Bradstreet: 0.67 [0.62, 0.71]	N/A
Powell [17] estimate (SE)	InfoUSA: 0.54 (0.04)	*InfoUSA: 0.44 (0.03)*	*InfoUSA: 0.32 (0.03)*	InfoUSA: 0.50 (0.03)	InfoUSA: 0.62 (0.03)	*InfoUSA: 0.38 (0.02)*	InfoUSA: 0.09 (0.01)	InfoUSA: 0.52 (0.04)	InfoUSA: 0.09 (0.01)
	Dun and Bradstreet: 0.46 (0.04)	Dun and Bradstreet: 0.29 (0.03)	Dun and Bradstreet: 0.22 (0.02)	Dun and Bradstreet: 0.38 (0.03)	Dun and Bradstreet: 0.48 (0.03)	Dun and Bradstreet: 0.27 (0.02)	*Dun and Bradstreet: 0.22 (0.01)*	Dun and Bradstreet: 0.61 (0.03)	*Dun and Bradstreet: 0.19 (0.01)*

Italic indicates statistically higher validity statistic as compared to the other data source

Standard errors and confidence intervals reported as originally reported in the cited papers

#### Locational accuracy

Locational accuracy was the third factor we considered. In both InfoUSA and Dun and Bradstreet, geocodes, or geographic coordinates (i.e., latitude and longitude), are provided for each record. Geocode quality depends on the precision of the match between the input address and the underlying road file. The match may be to the street address or to the centroid of larger administrative units, including ZIP + 4, ZIP, first two digits of the ZIP, city, or state.

Locational accuracy has been validated in two different ways: accuracy of the point location and accuracy of assignment to administrative units. Liese et al. [15] report that InfoUSA and Dun and Bradstreet perform similarly on both point location and accuracy of assignment. However, Liese et al. [16] found that including locational accuracy in an assessment of undercount of food stores and restaurants caused InfoUSA’s accuracy statistics to decline more than Dun and Bradstreet’s (which, without locational error considered, were worse than InfoUSA). As a result, the two business lists generally showed similar accuracy statistics when accounting for locational error. In the case of limited-service restaurants, InfoUSA continued to significantly underperform (97.3%, 95% CI 96.0, 98.5, undercount in InfoUSA vs. 67.3%, 95% CI 63.7, 70.9, in Dun and Bradstreet). One study examined locational accuracy by urbanicity and found that records in urban and suburban areas were geocoded more accurately than in rural areas in both InfoUSA and Dun and Bradstreet [15] (Table 3).

### Lessons learned for WAVES

When choosing which data source to buy, we considered all three key factors—count, classification, and locational accuracy—as well as systematic bias in each data source. In particular, we paid close attention to classification accuracy because we knew that the size of the dataset we expected to purchase would preclude attempts to reclassify individual records by hand. In general, InfoUSA tended to show slightly better count and classification accuracy statistics than Dun and Bradstreet, and both performed similarly with respect to locational accuracy. However, InfoUSA showed poor accuracy classifying limited service restaurants. While limited bias by neighborhood racial/ethnic composition, economic characteristics, and urbanicity was found in both InfoUSA and Dun and Bradstreet, there was no evidence that either source was consistently more biased than the other. Therefore, we purchased food store data from InfoUSA and restaurant data from Dun and Bradstreet. Depending on study questions and new information about the validity of commercial business lists, other teams may make different decisions.

### Maximizing purchased data quality

We pursued several strategies, first in purchasing and then in data cleaning, to optimize the validity of our study data, which are described below. Some steps were used regardless of the data vendor, while others were specific to a particular vendor, as noted below. The changes introduced by all data cleaning strategies were manually checked with a small random sample of records to confirm accuracy. In this way we ensured the highest quality retail food environment data possible given the limitations of the data source. We used retail food outlet data purchased for the years 2007–2014. These data years allowed for both 1- and 2-year lags in the retail food environment relative to the individual-level health outcome measures in our study.

#### Supplementing data by outlet name

Because validation studies showed lower sensitivity and PPV for both InfoUSA and Dun and Bradstreet data when accounting for misclassification by SIC code, our primary concern related to accuracy during the purchasing phase of the study was the failure to purchase desired outlet data due to inaccurate SIC code classification in the business lists. Therefore, in addition to purchasing each store or restaurant type by requesting all establishments within a list of SIC codes, we requested a record search by company name. The SIC code list was developed through an extensive literature review and in consultation with the business list sales representatives [8, 9, 16, 17, 19, 20, 24, 25]. The list of company names included national chains of supermarkets, pharmacies, convenience stores, general merchandise stores, and limited service restaurants, and was developed from lists of the largest chains of those establishment types (Table 5). All SIC codes and chain names by outlet type are available in Additional file 1. The chain name search helped ensure that we would receive records of chain outlets that had been inaccurately classified within the databases by SIC code. For example, if a McDonald’s record had an SIC code for full-service (which we did not purchase) rather than limited-service restaurant, it would be identified and purchased using the name search strategy.

**Table 5:** Sources for chain name lists

Business type	Source	Years
Supermarkets/grocery stores	Supermarket News Top 75 Retailers and Wholesalers	2010–2014
Convenience stores	Convenience Store News Top 100 Convenience Store Companies	2013
Pharmacies	Chain Drug Review Top 50 Chains (pharmacy dollar value and pharmacy count)	2013
General merchandise stores	Expert opinion	N/A
Limited service restaurants	National Restaurant News Top 200 (quick service and fast casual)	2007–2013
	Quick Service Restaurant Top 50	2007–2013

#### Reclassifying outlet types

In addition to failure to purchase data because of incorrect SIC classification, we were concerned about records within our dataset being identified as incorrect outlet types, so we developed an automated reclassification technique. The retail food outlet data purchased from InfoUSA contained establishments of various types, including supermarkets and grocery stores, convenience stores, pharmacies, liquor stores, and general merchandise stores. Records were initially given a provisional store type classification based on SIC code. The same list of chain names used for purchasing was applied to the data to identify records misclassified by SIC code by searching both the complete correct spelling and various versions of incorrect spellings and abbreviations in both the company name and corporate name data fields. Records identified as chains of a different type than the provisional classification were reclassified to a consistent type. Records purchased from Dun and Bradstreet that did not have a limited service SIC code but that were on the list of chain names were all reclassified as limited service restaurants.

#### Improving locational accuracy

Besides incorrect classification, validation studies indicated that accuracy of purchased data was lowered due to locational inaccuracies. Given the scope and resources of the study, it was infeasible to re-geocode all outlets across the 8 years. Thus, we evaluated and improved the provided geographic coordinates of records in two ways: screening out records based on the quality of geocoding and amending records with PO Boxes rather than street addresses. For the first locational improvement strategy, geocoding quality was determined based on codes provided by each company indicating precision of geocoding match. We only retained records that were geocoded to exact street address or ZIP + 4. In this way, we avoided clusters of stores at the centroids of larger administrative districts, which may have biased our findings.

The second locational improvement strategy dealt with records in the InfoUSA dataset between 2007 and 2010 that had PO Boxes listed in the address field. For establishments that were traceable through time using a business identification code and that had PO Boxes listed in the address field in some years and street addresses in other years, we used a “backcasting” method to improve the records. To do this, based on the assumption that businesses had not changed location in the intervening years, we assigned the geocode of the earliest year with a street address with acceptable geocoding accuracy to all prior years. For example, for a business with a PO Box address between 2007 and 2010 and a street address geocoded at the ZIP + 4 level in 2011, the 2011 geocode was backcast, or assigned, to the records between 2007 and 2010. The Dun and Bradstreet dataset did not have any records with PO Box addresses; therefore, this step was not required.

#### Deduplicating records

The final data improvement step was deduplicating records. Multiple incidences of records that potentially represented the same business locations were found in the databases. Duplications resulted from typographical errors in listings leading to records that appeared to be different, as well as records for multiple stores at the same location. Retail food outlet deduplication was accomplished for each outlet type separately.

Deduplication for supermarkets and grocery stores, pharmacies, convenience stores, and liquor stores was accomplished using two strategies: company name matching and address matching. For both strategies, records were identified as potential duplicates if they were for the same store type and in the same city, state and ZIP code. For the company name matching strategy, two records with identical company name fields were identified as duplicates if they had slight differences at the end of the address field (e.g., street suffix spelled out vs. abbreviated, unit numbers vs. no unit numbers). For the address matching strategy, records were identified as duplicates if they had an exact match in the address field and non-matching company names. This identified records with misspelled company names, and pairs of records where one record identified the business name and another identified the corporate name. This also identified pairs of records indicating two different stores of the same type operating out of the same location at the same time. The same technique using only the company name matching strategy was used for limited service restaurants because there are cases when multiple outlets of limited service restaurants operate out of the same location at the same time (e.g., Pizza Hut and Taco Bell combination locations).

General merchandise stores were found to regularly have multiple listings for the same location with both different names and different addresses. In part, this is because different departments in large general merchandise stores often had their own listings (e.g., Walmart Optical Center and Walmart Tire Center both within the same Walmart Supercenter). Because we were unable to reliably deduplicate by either name or address (because both name and address were different), we chose to deduplicate general merchandise stores geographically. A small pilot in several urban areas indicated that general merchandise stores of the same brand were unlikely to locate within one mile of each other (e.g., two Target stores in one mile or two Costco stores in one mile). Therefore, all general merchandise store records located within one mile of each other with the same corporate name were considered to be one store. We used geographic information system (GIS) software to merge all same store records to the geographic mean of all separate records [26].

## Results

### Supplementing data by outlet name

Due to the chain name search we requested in addition to SIC codes when purchasing, we acquired a more complete set of retail food outlet records. Column 1 in Table 6 shows the total number of records that were purchased from InfoUSA and Dun and Bradstreet; Column 2 is the number of records purchased by SIC code; and Column 3 shows the additional number of records purchased because of the chain name list. Without the name search, we would have missed 5.5% of records purchased from InfoUSA and 1.8% of records purchased from Dun and Bradstreet. Of the restaurant data purchased from Dun and Bradstreet, 63,162 records (2.9%) were identified as exact duplicate records by business identification number (D-U-N-S number).

**Table 6:** Purchased and final business counts for InfoUSA (food stores) and Dun and Bradstreet (restaurants), 2007–2014

	Purchased data			Final data		
	1	2	3	4	5	6
	Count N	By SIC code N (%)	By name N (%)	Count	Unused^a^	Percent unused
InfoUSA	2,847,339	2,690,245 (94.5)	157,094 (5.5)	2,341,030	506,309	17.5
Dun and Bradstreet	2,143,147	2,104,369 (98.2)	38,778 (1.8)	2,023,032	120,115	5.6

^a^Records were unused in the final dataset if they had insufficiently accurate geocoding, were duplicates, or were purchased in error

### Reclassifying store types

Following provisional classification of records by SIC codes, 18,924 food store records (0.7%) were reclassified using chain name lists from one type to another. The reclassification was primarily from pharmacies (n = 13,830) into general merchandise stores, with 1774 records reclassified from supermarkets/grocery stores to general merchandise stores, 2505 records from supermarkets/grocery stores to convenience stores, 30 records from supermarkets/grocery stores to pharmacies, 529 records from convenience stores to general merchandise stores, and 25 records from liquor stores to general merchandise stores. An additional 718 records purchased by name were classified into a store type without having first been assigned a provisional classification, including 85 records into general merchandise stores and 633 records into pharmacies. For restaurant records, 14,738 records (0.7%) were identified by name as full-service restaurants and 505 records (0.02%) were identified as convenience stores. These records were removed from the restaurants dataset. The first two rows of Table 7 show store type counts before and after reclassification by name.

**Table 7:** Store and restaurant counts before and after processing, overall and by store type, 2007–2014

	Supermarkets and grocery stores	Convenience stores	Pharmacies	Liquor stores	General merchandise stores	Limited service restaurants
Provisionally classified by SIC code	712,033	1,152,453	461,555	306,881	57,323	2,079,985
Reclassified by name	707,724	1,154,429	448,388	306,856	73,566	2,064,742
After cleaning for locational accuracy	643,306	1,038,993	424,842	286,261	69,592	2,023,032
After deduplication by name	624,845	977,178	408,979	280,473	N/A	N/A
After deduplication by address	624,700	977,165	408,935	280,473	N/A	N/A
Final count after cleaning and deduplication (excluding AK, HI)	621,343	972,735	407,270	278,895	60,787	2,023,032

General merchandise stores were deduplicated using geographic deduplication. Count changes in the final step are due to exclusion of records in Alaska and Hawaii

### Improving locational accuracy

The first strategy to improve locational accuracy, dropping observations with geocoding quality less specific than the ZIP + 4 level, reduced the incidence of clusters at the centroid locations of administrative units. Overall, this strategy eliminated 8.5% of records from the InfoUSA food stores dataset and 2.0% of records from the Dun and Bradstreet restaurant dataset. The second strategy backcasted geocodes of records with PO Box addresses. Between 2007 and 2010 7.8% of InfoUSA food store records had PO Boxes listed in the address field, compared to 0.3% of records between 2011 and 2014. Across the years 2007–2010, 7.5% of InfoUSA food store records were backcast from street or ZIP + 4 level geocodes. The remaining 0.5% of records with PO Boxes listed in the address field in 2007–2010 were dropped, as were records from 2011 to 2014 with PO Box addresses. As noted above, the Dun and Bradstreet restaurant data did not contain records with PO Boxes and therefore this step was not applied to Dun and Bradstreet restaurant data. The third row of Table 7 shows store type counts after cleaning for locational accuracy.

### Deduplicating records

The fourth and fifth rows of Table 7 show counts after each stage of deduplication. Deduplication by name was more effective than deduplication by address. For example, deduplication by name reduced the convenience store sample by 61,815 records, and deduplication by address only reduced the sample by a further 13 records (Table 7). Geographic deduplication for general merchandise stores reduced the sample by 12.6%. Overall, deduplication reduced the InfoUSA sample by 4.9%. Deduplication was not done at this step for Dun and Bradstreet data because of multiple limited service restaurants at the same location (see deduplicating records in “Methods” section).

### Overall impact

To understand the overall impact of this multistep data cleaning process, we looked at a variety of statistics. Purchased records were not included in the final dataset because they lacked sufficient geocoding accuracy, they were duplicates, or they were purchased in error. In the InfoUSA dataset of food stores, 17.5% of purchased records were unused and in the Dun and Bradstreet dataset of restaurants, 5.6% of records were unused (Table 6) (It is important to remember that these two data reduction numbers are not directly comparable because they represent different business types). The joint effect of the processing steps of reclassifying by name, improving locational accuracy, and deduplicating records was to reduce the InfoUSA dataset by 13.0% from the provisional classification (by SIC code) step and the Dun and Bradstreet dataset by 3.8% (Table 6).

We also compared counts of all supermarkets, grocery stores, convenience stores, pharmacies, liquor stores, general merchandise stores, and limited service restaurants combined in each census tract before and after data processing. Data processing resulted in a net change in outlet count in 74% (2007 data), 83% (2010 data) and 83% (2013 data) of census tracts in the continental US. Count changes in census tracts ranged from 6 more outlets in the census tract after processing to 134 fewer outlets after processing (data not shown). Increases in outlet count were due to improving locational accuracy by backcasting for records with PO boxes. Reductions in outlet counts were due to unused records from insufficiently accurate geocoding, deduplication, or records purchased in error that did not receive an outlet type classification either from SIC code or name.

## Discussion

This paper responds to the need for both strategies to improve retail food environment measurement and transparency in environmental characterization that will facilitate comparability between studies [27]. While the use of commercial business lists can be problematic for both validity and cost reasons, there are many studies for which they are a feasible solution to the problems associated with primary data or other types of secondary data. Additionally, because of limitations due to data costs and study scope (e.g., sizable geographic coverage, multiple years of data), it may only be financially feasible to buy from one company, even though some suggest that the combination of multiple databases produces more accurate results [20, 22]. In those cases, published validation results can be helpful in making the decision about which business list to use. However, the findings in various validation studies can be difficult to apply, and are not always directly comparable. Moreover, commercial business list data have recognized limitations and little information has been published about steps that can be taken to improve the quality of purchased data and the impact of these strategies.

**Fig. 1:**
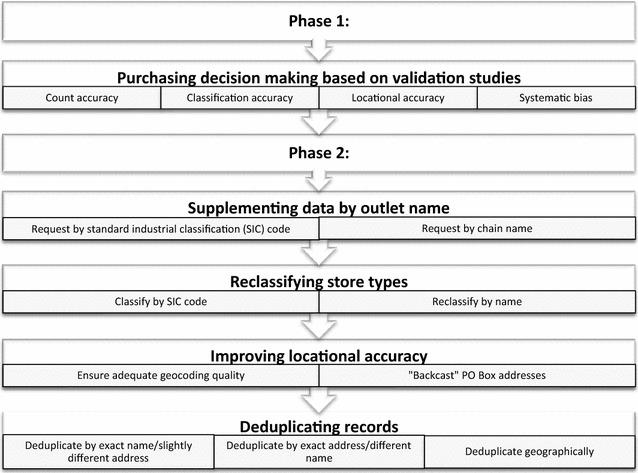
Best practices for commercial business list data purchasing and processing

Following our methods in WAVES, the included figure depicts best practices that can help to ensure the highest possible retail food environment data quality (Fig. 1). Our approach begins by reviewing results on count, classification and location accuracy as well as systematic bias in previously-conducted validation studies of InfoUSA and Dun and Bradstreet. Because of the aims of our study, we paid particularly close attention to classification accuracy. Next, the approach involves supplementing SIC code lists with business names when purchasing data, in an effort to capture records that had been incorrectly classified by SIC code in the dataset. Processing of the records involves three major steps: reclassifying store types by name, ensuring locational accuracy, and deduplicating records. Because these processing steps can all be automated, they can be applied in research studies of any size and including both contemporary and historic data. Our findings indicate that following these best practices has a significant impact on the dataset.

While the process we implemented for using the business list data appears to have improved the quality of the data, it does have limitations. There are certain classes of records that were not identified and thus could not be corrected. For example, we were not able to identify records of locations that have closed or wholly erroneous records (i.e., no associated establishment). Additionally, deduplication did not eliminate duplicate records if there were differences in both name and address fields, due to misspellings, abbreviations, or corporate vs. “doing business as” names. Similarly, this process did not impute missing records. Records may be missing across all years, or they may be missing only in some years. Records may be missing from the purchased list because they are improperly identified by SIC code and do not have a recognized chain name and so were not purchased, or they may be entirely missing in the business lists.

In conclusion, research on neighborhood retail food environments and health often necessitates the use of commercial business listings. However, the purchase and preparation of data from commercial business lists is complex. Decisions about which data to purchase are dependent on the study questions as well as available resources, and many types of validity and potential biases must be considered and weighed. After purchase, careful attention must be paid to the data in order to have the highest quality dataset possible, with this study suggesting several best practices that can be used in future studies to improve food environment measurement and transparency in reporting.

## Additional file

**Additional file 1.** Purchasing instructions for all business types.

## Abbreviations

PPV: positive predictive value
SIC codes: standard industrial classification codes
VA: Veterans Health Administration
WAVES: Weight And Veteran’s Environments Study
